# Supplementary material for: The landscape for HIV pre-exposure prophylaxis during pregnancy and breastfeeding in Malawi and Zambia: A qualitative study
Source: PLoS One. 2019 Oct 4;14(10):e0223487. doi: 10.1371/journal.pone.0223487 (PMC6777778; doi:10.1371/journal.pone.0223487)
Supplement: Attachment — Submitted filename: Response to reviewers.pdf [file pone.0223487.s001.pdf]

RESPONSE TO REVIEWERS

Editor's comments

Congratulations on a well conducted study and mostly an unbiased account of your diverse findings. A few suggestions to strengthen the readership interest:

1. If available, include recent HIV incidence and MTCT rates in pregnant and breastfeeding populations in these two countries (an indication for prioritising this population in PrEP policies and guidelines).

> *This has been included in the revised manuscript (lines 51-59). We have included data about HIV incidence among women of reproductive age, based on population-based surveys in Malawi and Zambia. We also include data from historical studies of HIV incidence during pregnancy and breastfeeding in these countries. Finally, we provide estimates from UNAIDS about the relative contribution of new maternal HIV infections to new infant HIV infections in the two target countries.*

2. In the Discussion, briefly discuss when Zambia and Malawi implemented the PMTCT programme, current status of the PMTCT programme, whether donor agencies primarily support the programme and your thoughts on how PrEP can be integrated into current PMTCT guidelines. Include discussions with HCW and policy makers if available.

> *Thank you for this suggestion. We have included a paragraph about the current status of the PMTCT programs in Malawi and Zambia, including their success in expanding antiretroviral therapy coverage. We discuss the concerns raised by policymakers about the prioritization between HIV prevention (PrEP) and treatment (antiretroviral therapy) in the context of donor-funded programs (lines 329-336). In the following paragraph (lines 338-353), we provide suggestions on how national HIV programs might integrate PrEP into existing maternal and child health platforms, including PMTCT.*

3. Lastly, transcript for PM607 is repeated. You could either find another transcript or consolidate your interpretations/application while citing the transcript once.

> *Thank you for flagging this repetition. Transcript PM607 is now only cited once.*

Reviewer #1

Thanks for the opportunity to review this manuscript. It is clearly written and interesting. Here are my comments:

Introduction:

1) Line 54 "Policymakers were identified from existing governmental technical working groups" Is this in both countries?

   Yes. We have added a phrase to indicate that this applies to both countries

Methods:

1) Please include approximate length of the interviews.

   Interviews lasted approximately one hour (line 100).

2) Please include the approach for participant selection.

Patients and their partners were recruited via a convenience sample. The eligibility and approach are described further in lines 71-81.

3) Could the authors describe a little more about the participant selection; was the intention to interview 39 women? Was age or parity a criterion? Why 7 men? Please give a little more detail.

The eligibility criteria and approach are described in lines 71-81. Our target accrual was 40 HIV-negative pregnant or breastfeeding women, up to 40 primary male partners (based on the index women's permission to recruit), 20 HCWs, and 20 policymakers. The sample was divided evenly across the two country sites (lines 81-83).

4) Please include the theoretical approach for analysis.

We have included additional text in the revised manuscript. In lines 61-64, it reads: "We used a qualitative descriptive approach in the design, data collection and analysis of this formative study [18, 19]. Our goal was to provide accurate accounting of events—and their meaning—from the individuals interviewed [18]. We drew from the basic tenets of naturalistic inquiry, with no specific commitment to a pre-defined theoretical framework [20]."

Results:

1) Could you add the dates to the title in Tables 1 & 2?

This has been added to the titles for Tables 1 and 2.

2) Were there any differences in sociodemographics by country?

Because of our small sample size, we did not further stratify the participating women and their male partners by country. We were concerned that, with 20 or fewer participants in any one category, differences would be difficult to interpret.

3) Please remove the participant ID numbers. This is not helpful to the reader.

These have been removed as requested.

4) Do we know the HIV status of the male partners?

Out of 14 male partners, 13 were reported to be HIV-negative. Only 1 partner had unknown HIV status (Table 2).

5) Would be nice to know the gender and age of the health care workers and policy makers. Is this possible?

We agree that this information could be helpful. Unfortunately, we did not collect this information for healthcare workers and policy makers for reasons of confidentiality. Because

these groups are small, there were concerns that such details could lead in accidental disclosure. This is now explained in lines 89-90.

Discussion:

1) Would be nice to discuss how this could be similar or different in different populations. What about younger women 15-18years? What about higher risk groups?

   In lines 340-342, we discuss the importance of screening and triage procedures for pregnant/breastfeeding women at elevated risk for HIV acquisition. Currently, there are few validated measures; however, it is acknowledged—including by respondents in this study—that such instruments could help to make PrEP services more efficient and sustainable.

2) Is there any data about PrEP in other groups of adults in Zambia or Malawi?

   Data about PrEP use in these countries remains limited. In Malawi, programs are only now beginning. In Zambia, pilot programs have been implemented for other key populations. These are now referenced in lines 365-367.

3) As noted in the limitations – there are PrEP programs already in place in most countries now, this is less interesting since its based on intention rather than on real experience.

   As noted in this comment, this limitation has been acknowledged within the paper. However, since the majority of countries have not implemented large-scale programs targeting pregnant and breastfeeding women specifically, we believe this remains a worthy contribution to the public health literature.
